# Pancreatic cancer subtyping - the keystone of precision treatment

**DOI:** 10.3389/fimmu.2025.1563725

**Published:** 2025-04-08

**Authors:** Zeyang Fan, Yao Xiao, Yan Du, Yan Zhang, Wence Zhou

**Affiliations:** ^1^ The Second Clinical Medical School, Lanzhou University, Lanzhou, China; ^2^ Department of General Surgery , The Second Hospital of Lanzhou University & The Second Clinical Medical School, Lanzhou University, Lanzhou, China

**Keywords:** pancreatic cancer, subtypes, genomics, transcriptomics, proteomics, metabolomics, immunomics

## Abstract

In recent years, the incidence and mortality rates of pancreatic cancer have been rising, posing a severe threat to human health. Tumor heterogeneity remains a critical barrier to advancing diagnosis and treatment efforts. The lack of specific early symptoms, limited early diagnostic methods, high biological complexity, and restricted therapeutic options contribute to the poor outcomes and prognosis of pancreatic cancer. Therefore, there is an urgent need to explore the different subtypes in-depth and develop personalized therapeutic strategies tailored to each subtype. Increasing evidence highlights the pivotal role of molecular subtyping in treating pancreatic cancer. This review focuses on recent advancements in classifying molecular subtypes and therapeutic approaches, discussed from the perspectives of gene mutations, genomics, transcriptomics, proteomics, metabolomics, and immunomics.

## 1 Introduction

Recent epidemiological data indicate that pancreatic cancer is a highly lethal disease, with a 5-year survival rate of approximately 13% at diagnosis, and it is gradually becoming one of the most common causes of cancer-related death (1). Pancreatic cancer causes over 400,000 deaths annually and has already become the third leading cause of cancer-related deaths worldwide (1). By 2030, it is projected to become the second leading cause of cancer-related mortality (2). Among pancreatic cancers, pancreatic ductal adenocarcinoma (PDAC) accounts for approximately 90% of cases (3).

The clinical management of pancreatic cancer currently relies on a four-tier staging system (resectable, borderline resectable, locally advanced, and metastatic) (4, 5). Apart from surgical resection combined with chemotherapy, no other approaches have been shown to significantly prolong patient survival (6). In fact, only 10%-15% of patients present with resectable disease at the time of diagnosis (5). Even among patients who undergo surgical treatment, the 5-year survival rate is only 20% (4), and 69%-75% of these patients eventually experience recurrence within two years, while 80%-90% relapse within five years (7). Despite advances in multidisciplinary treatment strategies, pancreatic cancer remains a systemic disease with no substantial improvement in prognosis (8).

Currently, two main factors contribute to the poor prognosis of PDAC. The first is the structural characteristics of PDAC itself: its complex tumor composition and architecture create a hypoxic microenvironment while isolating the tumor mass from external interactions, leading to drug resistance. The second factor is the intrinsic heterogeneity of pancreatic cancer, which includes intertumoral and intratumoral structural heterogeneity, molecular heterogeneity, subtype interconversion, and subtype transitions during disease progression (9). Therefore, addressing tumor heterogeneity to develop personalized treatments for individual patients has become a major focus of current research. This approach has already been validated in other solid tumors, such as targeting human epidermal growth factor receptor-2 (*HER2*) to treat *HER2*-overexpressing breast cancer. However, molecular subtyping of pancreatic cancer remains in its infancy, and clinically actionable subtypes for guiding therapeutic decisions have yet to be defined (5, 10).

Translating the latest advances in the molecular characteristics of pancreatic cancer into targeted therapies is an active area of ongoing research (4). In the coming years, the development of drugs designed to target specific molecular subtypes and associated pathways of pancreatic cancer is expected to make significant contributions to personalized and subtype-specific treatments. These novel drugs may be used in combination with certain first-line therapies to reduce mortality, extend overall survival (OS), and potentially address resistance to some first-line treatments. Subtyping pancreatic cancer based on different criteria holds potential clinical applications, as precision therapy focuses on distinguishing specific groups of patients with unique characteristics and treating them by targeting their specific molecular targets (10).

**Figure 1:**
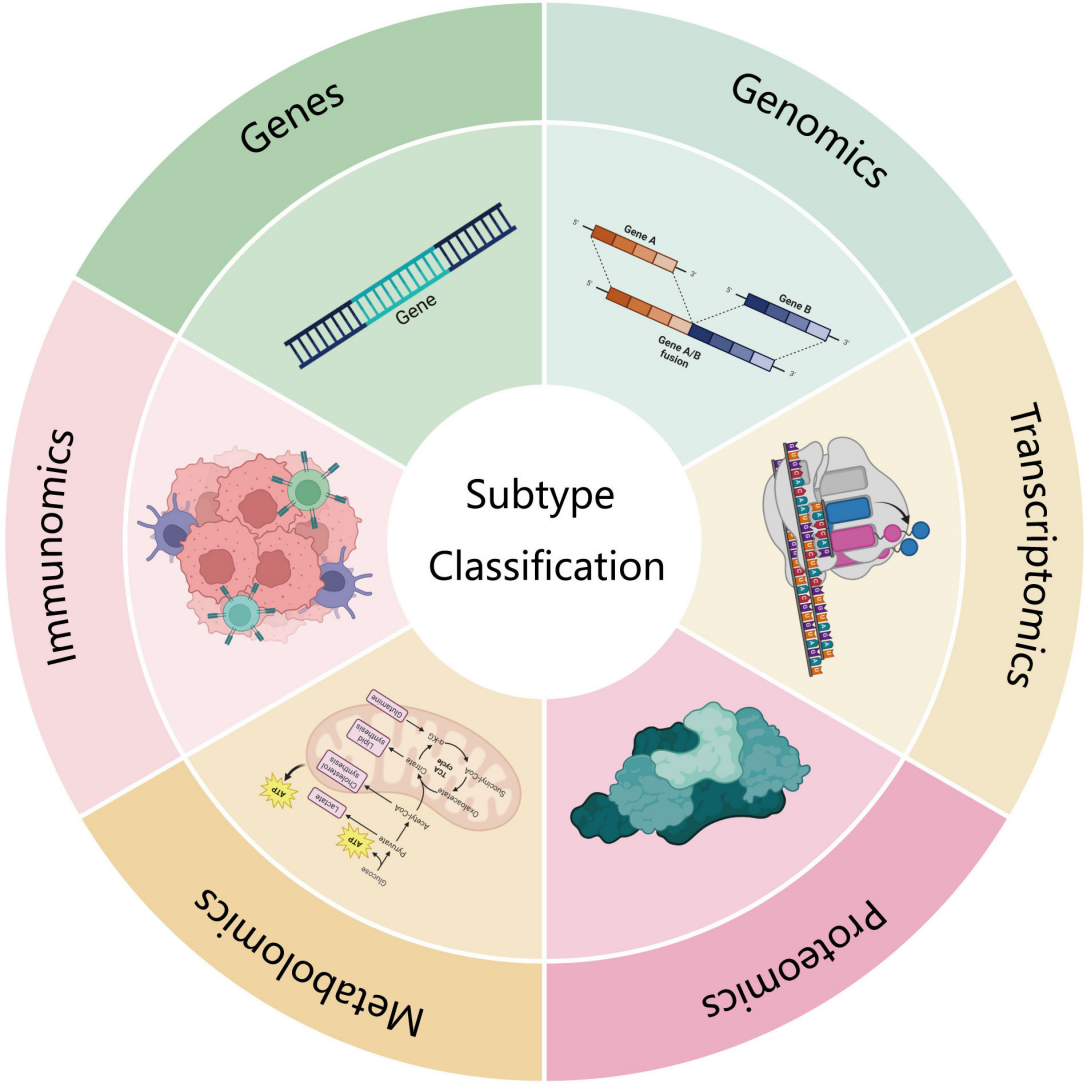
This review looks at several aspects of gene mutation, genomics, transcriptomics, proteomics, metabolomics and immunomics.

Currently, the classification criteria for pancreatic cancer subtypes are highly diverse. This review aims to summarize and discuss the most recent and influential subtyping strategies from multiple perspectives, including gene mutations, genomics, transcriptomics, proteomics, metabolomics, and immunomics (**Figure 1**, **Table 1**). Additionally, it provides a more comprehensive discussion of metabolomics and immunomics, which have been relatively underexplored in previous reviews.

**Table 1:** Major subtype classification.

Classification	Author	Samples	Subtype	Ref
Genomics	Waddell, Pajic, Grimmond et al	100PDAC	Stable Locally rearranged Scattered Unstable	(26)
	Connor, Denroche, Gallinger et al	discovery cohort comprised 160 PDAC cases from 154 patients replication cohort comprised 95 primary PDAC.	Age-related DSBR MMR Signature 8	(52)
Transcriptomics	Collisson, Sadanandam, Gray et al	PDAC from UCSF and several microarray datasets Human and mouse cell lines	Classical Exocrine-like QM-PDA	(54)
	Moffitt, Marayati, Yeh et al	145 primary and 61 metastatic PDAC	classical basal-like	(56)
	Noll, Eisen, Sprick et al	8 PACO cell systems and their PT and DT allografts	KRT81+HNF1A− KRT81−HNF1A+ KRT81−HNF1A−	(58)
	Bailey, Chang, Grimmond et al	456 PDAC	pancreatic progenitor squamous ADEX immunogenic	(59)
	Sivakumar, de Santiago, Markowetz et al	242 pancreatic cancers from ICGC 178 pancreatic cancers from TCGA 5 pancreatic cancer related datasets from GEO	Hedgehog/Wnt NOTCH cell cycle	(66)
	Puleo, Nicolle, Maréchal et al	309 paraffin embedded PDAC samples	pure classical immune classical pure basal like stroma activated desmoplastic	(61)
	Mueller, Engleitner, Rad et al	38 PK mice 19 PanIN patients	C1/C2	(67)
	Chan, Kim, Notta et al	317 PDAC	Basal-like-A Basal-like-B Hybrid Classical-A Classical-B	(43)
	Birnbaum, Begg, Liss et al	28 PDAC	C1/C2/C3/C4	(65)
	Shi, Li, Gao et al	84 pancreatic cancer-like organs	Classical-like Basal-like Classical-Progenitor Glycomet	(70)
	Kim, Leem, Park et al	17 PDAC	Ep_TRIM54 Ep_KRT6A Ep_PIFO Ep_MSMB Ep_VGLL1	(72)
Proteomics	Zhao, Zhao, Yan	1200 PDAC	L1/L2/L6	(87)
	Law, Lagundžin, Woods et al	68 PDAC	Metabolic Progenitor-like Proliferative Inflammatory	(88)
	Son, Kim, Kim et al	225 PDAC	stable exocrine-like activated ECM remodeling	(89)
	Tong, Sun, Ding et al	217 PDAC	S-I/S-II/S-III	(90)
	Hyeon, Nam, Lee et al	196PDAC	TS1/TS2/TS3/TS4/IS1/IS2	(92)
Metabolomics	Daemen, Peterson, Evangelista et al	38 PDAC	reduced proliferative capacity glycolytic lipogenic	(94)
	Karasinska, Topham, Schaeffer et al	325 PDAC	quiescent glycolytic cholesterogenic mixed	(96)
	Li, Du, Zhang et al	20 PAAD and 10 normal tissues	quiescent pyruvate GG mixed	(99)
	Li, Tang, Jin et al	28 PDAC	glucomet-PDAC lipomet-PDAC	(101)
Immunomics	Knudsen, Vail, Witkiewicz et al	109 PDAC	hot cold mutationally cold mutationally active	(105)
	Wartenberg, Cibin, Karamitopoulou et al	110 PDAC	immune escape immune rich immune exhausted	(106)
	Danilova, Ho, Yarchoan et al	152 PAAD	PD-L1+/CD8high PD-L1+/CD8low PD-L1-/CD8high PD-L1-/CD8low	(107)
	de Santiago, Yau, Sivakumar et al	353 PDAC	innate immune T cell dominant tumor dominant	(108)
	Hwang, Jagadeesh, Regev et al	43 PDAC	treatment-enriched squamoid-basaloid classical	(111)
	Tong, Sun, Ding et al	217 PDAC	Im-S-I/Im-S-II/Im-S-III/ Im-S-IV/Im-S-V	(90)

## 2 Subtype classification

### 2.1 Gene mutation and applications

**Figure 2:**
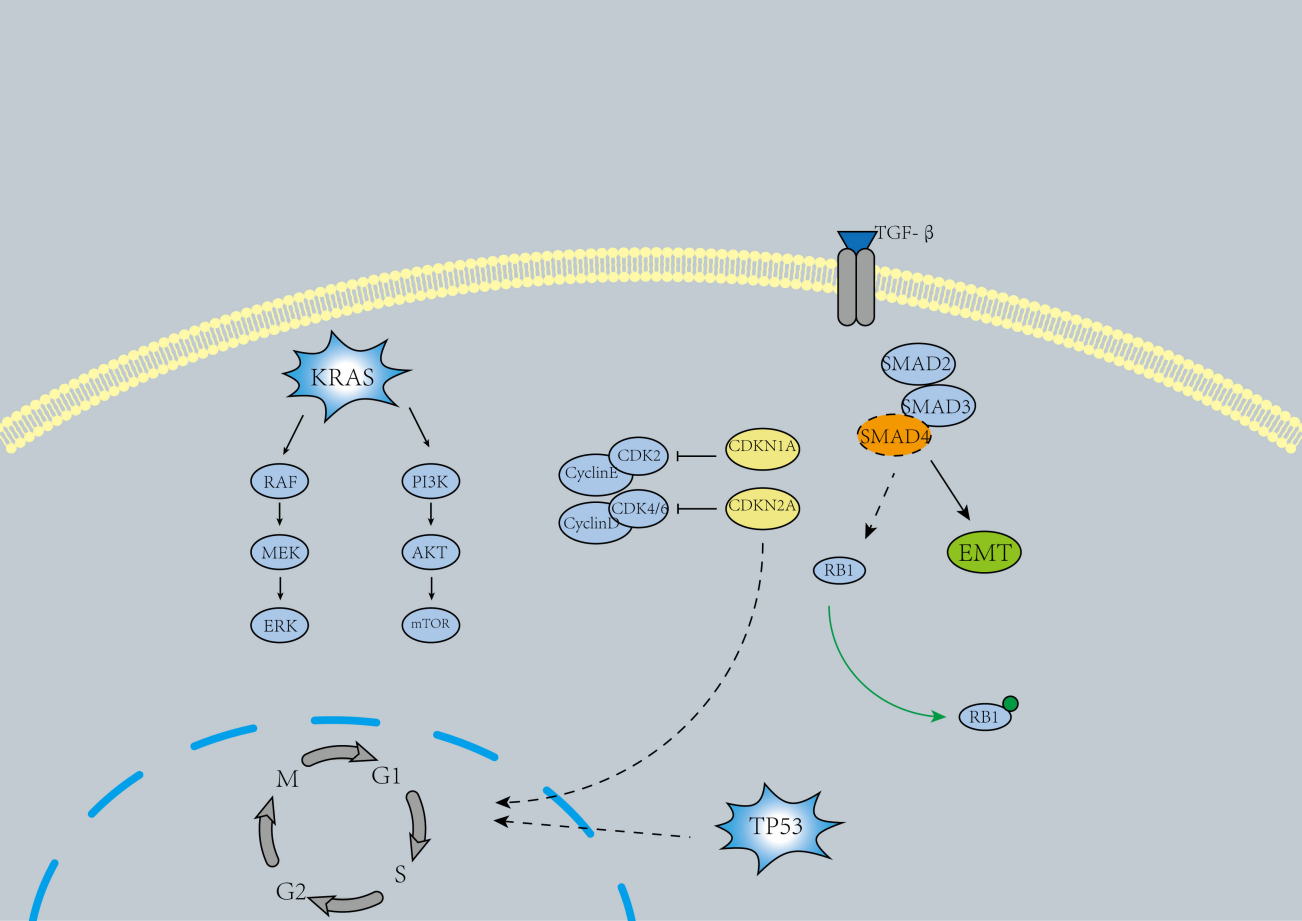
A mechanism map of the major pancreatic cancer causing genes.

*KRAS* is the most commonly mutated oncogene in pancreatic cancer and represents one of the earliest alterations observed in pancreatic intraepithelial neoplasia (Pan IN) (**Figure 2**), where *KRAS* mutations lead to the activation of downstream effectors, driving various pro-tumorigenic processes (11). Approximately 90% of pancreatic cancer patients harbor *KRAS* mutations (12). While *KRAS* was previously considered undruggable, the past decade has witnessed the emergence of several promising molecular therapies targeting *KRAS*. These include MRTX1133 (a *KRAS^G12D^* inhibitor) (13), RMC-6236 (14), ASP3082 (15), and BI1701963 (a pan-*KRAS* SOS1 inhibitor) (16). Notably, targeted therapies for the rare *KRAS^G12C^* mutant, such as Sotorasib, have shown therapeutic potential (17). Some researchers have shifted their focus to downstream molecules of *KRAS*, such as *EGFR*, *MEK*, and *PI3K*. However, results indicate that most *EGFR* and *MEK* inhibitors have not significantly improved patient outcomes (18). Interestingly, a recent study demonstrated that *EGFR* inhibition may provide tangible benefits in a selected subgroup of *KRAS* wild-type PDAC patients (19). Therefore, multiple drug combinations and further exploration of *KRAS* and its downstream signaling therapies may remain a hotspot for future research.

In pancreatic cancer, the inactivation of tumor suppressor genes *TP53*, *SMAD4*, and *CDKN2A* is another major oncogenic driver (4)(**Figure 2**). Inactivating mutations of *TP53* are identified in 50%-74% of pancreatic cancers (5, 18). Similar to *KRAS*, *TP53* mutations arise in Pan IN lesions and accumulate over time, ultimately driving the progression to pancreatic ductal adenocarcinoma (PDAC) (20). The primary oncogenic mechanism of *TP53* inactivation involves defective DNA damage recognition and the prevention of cell cycle arrest (4, 5).*TP53* reactivators include Cys-targeting compounds such as APR-246 (21), the compound ATO (22), and the antiparasitic drug sodium stibogluconate (SSG) (23). However, the applicability of these reactivators in pancreatic cancer treatment remains uncertain. Ongoing clinical trials may shed light on their potential to improve the prognosis of patients with *TP53* mutations.

The loss of *SMAD4* expression occurs in the late stages of PDAC tumor progression (24). Approximately 31%–38% of individuals with pancreatic cancer harbor *SMAD4* mutations, which are frequently lost through homozygous deletions or mutations. This results in the weakening of *SMAD4*-dependent inhibitory effects of transforming growth factor-β (TGF-β), thereby enhancing non-canonical TGF-β signaling and promoting pro-tumorigenic responses (25, 26). The loss of *SMAD4* is associated with disease metastasis (27). Disruption of the TGF-β-*SMAD4* signaling pathway in PDAC may induce epithelial-mesenchymal transition (EMT) (28). Cancer-associated fibroblasts (CAFs) secreting TGF-β may promote the proliferative phenotype of transformed PDAC cells, contributing to the heterogeneity of PDAC (29). In some studies, drugs targeting TGF-β, such as NIS793 (30) and Vactosertib (31), have shown promising efficacy.

In 46%–60% of pancreatic cancers, inactivating mutations of *CDKN2A* have been detected (4). The inactivation of *CDKN2A* is primarily caused by homozygous deletions, hypermethylation, or mutations combined with the loss of the wild-type allele, leading to dysregulation of the cell cycle in cancer cells (25, 32). The combined use of *CDK4/6* inhibitors and *ERK*-*MAPK* inhibitors may be effective for patients with *CDKN2A* and *KRAS* co-mutations (33).

Recent studies have identified several novel mutation/variant genes with frequencies below 20%, including *KDM6A*, *RAC1*, *RNF43*, *ARID1A*, *BRAF*, *TGFBR2*, *MAP3K21*, *SWI*/*SNF*-related, matrix-associated, *SMARCA4*, *ACVR2A*, *ACVR1B*, *NRAS*, *FAM133A*, *ZMAT2*, and *STAT3* (32, 34–37).PDAC is also associated with germline and somatic mutations in the homologous recombination repair pathway, including *BRCA2*, *ATM*, *BRCA1*, and *PALB2* (38). Individuals carrying *BRCA* germline mutations have a significantly increased risk of developing pancreatic cancer (39). Tumors with homologous recombination deficiencies due to *BRCA1*/*2* mutations exhibit heightened sensitivity to poly (ADP-ribose) polymerase (PARP) inhibitors (40). A pivotal phase 3 randomized trial demonstrated that PARP inhibitors can prolong progression-free survival in patients with *BRCA1/2* mutations (40).

Recent studies on MTAP deletion mutations may provide new ideas for pancreatic cancer treatment. MTAP deletion plays a crucial role in pancreatic cancer research, with approximately 20–30% of pancreatic cancers exhibiting this genetic loss. This deletion is closely associated with poor patient prognosis. Regarding sensitivity to PRMT5 inhibitors, MTAP deletion renders cancer cells more susceptible to these inhibitors. This increased sensitivity is attributed to metabolic reprogramming induced by MTAP loss, which enhances glycolysis and *de novo* purine biosynthesis, thereby increasing cellular dependence on PRMT5. PRMT5 inhibitors may suppress cancer cell growth by targeting these processes. Combination treatment strategies have shown promise for MTAP-deficient pancreatic cancer. For instance, the combined use of 2-deoxy-D-glucose (2-DG) and L-alanosine has demonstrated synergistic lethality against MTAP-deficient pancreatic cancer cells. Furthermore, clinical trials combining PRMT5 inhibitors with agents such as 5-azacitidine and pembrolizumab may enhance therapeutic efficacy, paving the way for new treatment strategies for pancreatic cancer (41, 42).

Overall, the current drugs directly targeting *KRAS*, *TP53*, *SMAD4*, and *CDKN2A* have shown limited efficacy. However, further studies are needed to evaluate the effectiveness of drugs targeting the upstream and downstream factors of these genes, which is expected to become a major research focus in the coming years. Additionally, designing drugs based on low-frequency mutated genes, such as *BRCA* and *STAT3*, may offer promising and feasible approaches for pancreatic cancer treatment. In summary, specific therapeutic strategies targeting different mutated gene subtypes in pancreatic cancer require further in-depth investigation.

### 2.2 Genomics subtyping and applications

It is well known that the accumulation of genomic aberrations in tumors leads to the classification of different genomic subtypes and contributes to disease heterogeneity. This heterogeneity arises from the persistent genomic instability during tumor progression (43). Structural variations (SV) are a specific category of chromosomal alterations that can induce various gene changes, including deletions, rearrangements, amplifications, and fusions. These changes have significant biological implications and potential pathogenic associations at the molecular genetic level, playing a crucial role in understanding the mechanisms of tumorigenesis. Early studies have demonstrated the presence of multiple gene alterations caused by chromosomal structural variations in PDAC (44). Most structural variations are intra-chromosomal and can be classified into seven types: intra-chromosomal rearrangements, deletions, duplications, tandem duplications, inversions, fold-back inversions, and amplified inversions. Inter-chromosomal translocations are less common (26).

In 2015, Waddell et al. conducted whole-genome sequencing of 100 PDAC samples (26). Their study defined four pancreatic cancer subtypes: Stable, Locally Rearranged, Scattered, and Unstable. The Stable subtype accounted for 20% of the samples and typically exhibited widespread aneuploidy. These tumor genomes contained fewer than 50 SV events and were often associated with mitotic defects (26). The Locally Rearranged subtype comprised approximately 30% of the samples. About one-third of these genomes displayed copy number amplifications in known oncogenes, such as *KRAS*, SOX9, and *GATA6*, along with therapeutic targets like *ERBB2*, *CDK6*, *MET*, *PIK3CA*, and *PIK3R3* (45–49). The remaining locally rearranged genomes involved complex genomic events such as breakage–fusion–bridge cycles, chromothripsis, and ring chromosomes (26). The other two subtypes were the Scattered subtype (<200 SV events) and the Unstable subtype (>200 SV events), accounting for 36% and 14% of the samples, respectively. The Unstable subtype indicated defective DNA maintenance, which might render these tumors sensitive to DNA-damaging agents (50). Additionally, the Unstable subtype was associated with deleterious mutations in *BRCA1*, *BRCA2*, and *PALB2*, as the unstable genomes tend to recruit patients with *BRCA1* or *BRCA2* mutations (5). Current *PARP* inhibitor trials recruit patients based on *BRCA1* and *BRCA2* germline deficiencies, and these patients may exhibit susceptibility to platinum-based drugs and PARP inhibitors (26). Other chromosomal stability maintenance genes, such as *XRCC4* and *XRCC6*, have also been detected in the Unstable subtype or tumors with *BRCA*-mutated features (51). These findings suggest that the Unstable subtype may be a suitable candidate for precision therapies involving platinum-based drugs and *PARP* inhibitors.

In 2017, Connor et al. (52) performed whole-genome sequencing on 154 patients and combined their data with samples from 95 pancreatic cancer patients in the ICGC cohort. They proposed classifying PDAC into four subtypes: Age-related, Double-Strand Break Repair (DSBR), Mismatch Repair (MMR), and Unknown Etiology (Signature 8). The Age-related subtype arises from the gradual accumulation of damage during cell division. DSBR is primarily caused by defects in homologous recombination repair (HRR) of double-strand breaks. This subtype is associated with enhanced local anti-tumor immunity, where infiltrating CD8+ T cells show increased cytolytic activity, accompanied by increased expression of co-regulatory molecules (*CTLA-4*, *PD-L1*, *PD-L2*, and *IDO-1*). This scenario is similar to the response of melanoma to checkpoint inhibitors, suggesting that this subtype may respond to immunotherapy (53).MMR arises from defects in DNA mismatch repair, and its characteristics are similar to those of DSBR. As for the Unknown Etiology subtype, its origin remains poorly understood. Although some studies have suggested that smoking may be its cause, the data from Connor et al.’s research could not substantiate this epidemiological link.

With the continuous advancement of genomic analysis technologies in both depth and breadth, more refined and comprehensive genomic-based classifications will likely emerge in the future. These classifications will provide strong evidence and guidance for developing disease treatment plans, facilitating the transition from traditional empirical treatment models to precision medicine based on genomic profiling.

### 2.3 Transcriptomics subtyping and applications

Although we have summarized the genes and genomic phenotypes of pancreatic cancer, it is clear that these findings do not fully capture the entire spectrum of pancreatic ductal adenocarcinoma (PDAC), and their therapeutic efficacy remains limited. Given that various cellular processes can influence gene expression, screening for differentially expressed transcripts can aid in better identifying potential therapeutic targets for PDAC. Over years of research, several classification schemes for transcriptional subtypes have been published, and we will discuss some of the key classifications and their associated therapeutic applications.

In 2011, Collisson et al. proposed classifying pancreatic cancer into three subtypes: classical, quasi-mesenchymal (QM-PDA), and exocrine-like, each with its distinct characteristics (54). The classical subtype exhibits high expression of adhesion-related genes and epithelial genes, along with high *GATA6* expression, and is sensitive to erlotinib. The QM-PDA subtype shows high expression of stromal-related genes and is sensitive to gemcitabine. In the exocrine-like subtype, tumor cell-derived digestive enzyme genes are expressed at relatively high levels.

In 2014, Kim et al. identified three subtypes of pancreatic cancer. For subtype 1, the enriched pathways are closely related to the immune system, including hematopoietic cell lineage, cytokine-cytokine receptor interactions, and calcium signaling pathways. This subtype is associated with a high R0 resection rate and better prognosis. Subtype 2’s enriched pathways are linked to fatal diseases like pancreatic cancer, renal cell carcinoma, and chronic myelogenous leukemia, and are often associated with poor prognosis. Subtype 3, which had a smaller sample size, showed gene overlap with Collisson et al.’s exocrine-like subtype through gene enrichment analysis (55).

In 2015, Moffitt divided pancreatic cancer into “classical” and “basal-like” subtypes (56). The classical subtype exhibited characteristics similar to the classical subtype defined by Collisson et al., with high expression of *GATA6*, which serves as a key marker to distinguish advanced pancreatic cancer classical subtypes from basal-like subtypes (57). The basal-like subtype was characterized by high expression of genes related to cadherins and keratins, along with high *KRAS^G12D^* expression. This subtype is typically associated with poorer prognosis but shows a better response to adjuvant therapy compared to the classical subtype. Moffitt also identified two stromal subtypes: “normal” and “activated.” The “normal” stromal subtype was marked by elevated expression of markers such as pancreatic stellate cells, smooth muscle actin, vimentin, and desmin (*ACTA2*, *VIM*, *DES*). Patients with this type of stroma typically had a better prognosis. The “activated” stromal subtype was characterized by the expression of macrophage-related genes, such as integrin *ITGAM* and chemokine ligands *CCL13* and *CCL18*, as well as other genes like the secreted protein *SPARC* and *WNT* family members *WNT2* and *WNT5A*, indicating its significant role in promoting tumor growth. Interestingly, Moffitt et al. found a high overlap between the genes expressed by basal-like tumors and stromal subtypes and the QM-PDA genes proposed by Collisson et al.

In 2016, Noll et al. used an immunohistochemical classification based on markers *KRT81* and *HNF1A* to classify pancreatic cancer (58). The classification included the following subtypes: *KRT81*+*HNF1A*− for the QM-PDA subtype, *KRT81*−*HNF1A*+ for the exocrine-like subtype, and *KRT81*−*HNF1A*− for the classical subtype. In this study, the exocrine-like subtype was found to be resistant to paclitaxel tyrosine kinase inhibitors due to the expression of CYP3A5.

In 2016, Bailey et al. analyzed 96 tumors with over 40% epithelial content and identified four subtypes: pancreatic progenitor, squamous, aberrantly differentiated endocrine exocrine (ADEX), and immunogenic (59). The squamous subtype was associated with mutations in *TP53* and *KDM6A*, and it exhibited a series of biological phenomena, including inflammation, hypoxic response, metabolic reprogramming, activation of the TGF-β signaling pathway, MYC pathway activation, autophagy, and upregulation of *TP63*ΔN and its target genes. This subtype was also closely related to hypermethylation and consistent downregulation of genes controlling pancreatic endodermal cell fate. The pancreatic progenitor subtype was linked to the expression of early pancreatic differentiation genes and showed upregulation of genes associated with fatty acid oxidation, steroid hormone biosynthesis, drug metabolism, and mucin O-linked glycosylation. ADEX played a significant role in the terminal differentiation phase of the pancreas, characterized by the upregulation of endocrine-exocrine differentiation genes. The immunogenic subtype was closely related to immune infiltration, particularly with infiltrating B and T cells, suggesting potential sensitivity to immune modulators. Notably, except for the immunogenic subtype, the other three subtypes defined in this study overlapped with those proposed by Collisson et al. Specifically, the “quasi-mesenchymal” subtype in Collisson’s study was renamed “squamous” in this research, the “classical” subtype became “pancreatic progenitor,” and the “exocrine-like” subtype was renamed ADEX. The existence of ADEX/exocrine-like subtype is still debated, with some theories suggesting it may be due to contamination by surrounding pancreatic tissue (56, 60–62), but some studies support its existence (63–65). Collisson et al. did not find evidence of the exocrine-like subtype in human and mouse cell lines, but it was observed in microanatomical samples (54).

In 2017, Sivakumar et al. combined the three biological processes regulated by *KRAS* with the classification system proposed by Bailey et al. Their research revealed several important findings. In squamous subtype samples, there was an overexpression of the Hedgehog/Wnt pathways, along with an accumulation of M2 macrophages. Despite the squamous subtype having the poorest prognosis, emerging evidence suggests that targeted therapies could apply to this subtype, offering the potential for improved treatment outcomes. In the immunogenic subtype, cell cycle processes were overexpressed in the samples. However, an interesting paradox arose: patients in this subtype exhibited almost no noticeable immune activity. This contradiction challenges the conventional understanding of immune-related subtypes and emphasizes the need for further research to explore the underlying mechanisms, which could provide deeper insights into the biological features of this subtype. For the ADEX samples, there was an overexpression of the Notch pathway. Furthermore, the study discovered a positive correlation between immune therapy targets, such as programmed cell death protein 1 (*PD-1*) and cytotoxic T lymphocyte-associated protein 4 (*CTLA4*), and Notch pathway activity, along with an enrichment of CD8+ T cells. These findings suggest that patients in the Notch group may be more suitable for immune therapy (66).

In 2018, Puleo et al. conducted an RNA chip analysis on 309 paraffin-embedded samples, integrating the tumor microenvironment and epithelial components of tumors to distinguish five subtypes (61). The pure classical subtype they defined is composed of classic tumors with both normal and activated stroma, as defined by Moffitt et al. The activated stroma refers to the presence of fibroblasts in an activated state, which undergoes phenotypic changes to become myofibroblasts. This transformation process imparts unique histological and cellular characteristics to the pure classical subtype. The immune classical subtype is composed of classic tumors and normal stroma, as identified by Moffitt et al., which gives this subtype its distinct features. The pure basal-like subtype, defined by Moffitt et al., consists of basal tumors and activated stroma. It is characterized by the absence of cellular stroma and the occurrence of tumor metastatic spread, reflecting the subtype’s unique biological behavior and providing a key entry point for subsequent studies on tumor metastasis mechanisms and the development of targeted therapeutic strategies. The stroma-activated subtype is composed of basal or classical tumors and activated stroma, which is described by Moffitt et al., reflecting the complex and diverse interactions between different tumor cell types and stroma components during tumorigenesis and progression. The desmoplastic subtype mainly consists of basal or classical tumors and normal stroma which is mentioned by Moffitt et al., and is notably characterized by low tumor content and high expression of vascularized stromal components (such as elastin), along with the highest degree of immune cell infiltration. Moreover, they observed a significant correlation between the expression of *MET* and nuclear *GLI1* with the stroma-activated and pure basal-like subtypes, suggesting that the MET and Hedgehog signaling pathways are activated in these subtypes. Additionally, human equilibrative nucleoside transporter 1 (*hENT1*) is expressed at relatively high levels in the classical subtype (including pure classical and immune classical), and since *hENT1* is a marker for gemcitabine sensitivity, this suggests that the classical subtype may be more sensitive to gemcitabine. The expression of *CTLA4* is higher in the immune classical and desmoplastic subtypes, which makes these two subtypes potentially more suitable for anti-*CTLA4* therapy. Furthermore, all other subtypes, except for the classical subtype, exhibit high expression of relevant immune checkpoints, indicating that these subtypes may be suitable for immune checkpoint inhibition therapy. These findings provide an important theoretical basis for the application of different therapies in various tumor subtypes and for optimizing treatment plans, significantly contributing to the advancement of personalized immunotherapy for pancreatic cancer.

In 2018, a study by Mueller et al. classified pancreatic cancer into two subgroups, C1 and C2 (67). C1 exhibits distinct epithelial-mesenchymal transition (EMT) characteristics, which are closely associated with the high expression of *KRAS^G12D^* and Ras-related transcriptional programs. In contrast, C2 is characterized by the high expression of epithelial differentiation genes. At the cellular morphology level, all C1 cell lines display mesenchymal cell characteristics, while C2 cell lines present typical epithelial cell morphology, creating a clear contrast between the two.

In a 2020 study, Dijk et al. classified PDAC into four subtypes: secretory, epithelial, compound pancreatic, and mesenchymal (64). The secretory subtype exhibits enrichment in both the endocrine and exocrine functions of the pancreas. The epithelial subtype is characterized by high expression of mitochondrial components and ribosomal-related features. The mesenchymal subtype displays characteristics associated with epithelial-mesenchymal transition, stromal interactions, and TGF-β signaling. The compound pancreatic subtype shares some similarities with the previously published classical subtype and ADEX/exocrine-like subtype, but enrichment analysis revealed that it more closely resembles the mesenchymal subtype. These findings are likely related to tumor heterogeneity and lay the foundation for further investigation into the complex and diverse mechanisms of intratumoral heterogeneity, as well as the development of more targeted diagnostic and therapeutic strategies.

In 2020, Chan et al. performed sequencing analysis on 248 purified primary and metastatic pancreatic cancer epithelial cell samples, identifying five subtypes: “Basal-like-A,” “Basal-like-B,” “Hybrid,” “Classical-A,” and “Classical-B” (43). The development of Classical-A/B tumors was associated with *GATA6* amplification and complete *SMAD4* loss, whereas Basal-like-A/B tumors were strongly correlated with complete *CDKN2A* loss and an increased frequency of *TP53* mutations. Metastatic basal-like tumors were often enriched with mutated *KRAS* and exhibited greater resistance to chemotherapy. Among these, Basal-like-A tumors demonstrated poor response to gemcitabine-based and mFOLFIRINOX chemotherapy regimens. Therefore, distinguishing the basal-like-A subtype from the basal-like subtype identified by Collisson et al. allows for a more precise prediction of chemotherapy sensitivity across subtypes. These features are of significant importance both for deepening the understanding of tumor biology and for identifying potential targeted therapies specific to each subtype (5). Furthermore, single-cell RNA sequencing of tumors from 15 patients revealed the coexistence of basal-like and classical expression features within individual tumors, providing direct evidence of intratumoral heterogeneity.

In 2021, Birnbaum et al. employed LCM and RNA-seq analysis to identify four cancer cell subtypes (C1–C4) and three peritumoral stromal subtypes (S1–S3), among which S1 was associated with better prognosis when paired with C1 and C2 subtypes (65). The C1 subtype was linked to genes involved in protein folding and leukocyte chemotaxis, whereas the C2 subtype showed a strong association with gene programs essential for pancreatic endocrine cell development and neuronal membrane signaling. The C3 subtype was related to nucleotide biosynthesis and protein translation regulation, while the C4 subtype was associated with oncogenic signaling pathways, highlighting its critical role in tumorigenic signaling mechanisms. The S1 subtype was related to developmental and cell differentiation pathways, S2 was linked to antigen processing and presentation, and S3 was associated with phospholipid synthesis and macromolecule modification. Further analysis revealed that the C1 and C3 subtypes correlated with the classical/pancreatic progenitor subtype, the C2 subtype aligned with the ADEX/exocrine-like subtype, and the C4 subtype was associated with the squamous/basal-like/quasi-mesenchymal subtype. Additionally, the S1 and S2 subtypes exhibited enrichment of normal and activated stromal subtypes, respectively. These intricate associations provide valuable insights into the biological heterogeneity of tumors, the interplay between subtypes, and the foundation for developing precise therapeutic strategies.

Espinet et al. classified pancreatic cancer into MC1 and MC2 subtypes, which were found to be associated with IFN expression, suggesting that effective inhibition of intrinsic interferon signaling could serve as a potential therapeutic approach for targeting these tumors (MC1) with minimal side effects on normal cells (68).

Ju et al. categorized pancreatic cancer into aggressive and moderate subtypes. The aggressive subtype was associated with pathways overlapping with DNA damage repair (DDR) mechanisms, including DNA replication, homologous recombination, mismatch repair, and upregulation of the P53 signaling pathway. This finding suggests that targeting repair proteins involved in DDR mechanisms may be a viable therapeutic strategy. In contrast, the moderate subtype exhibited upregulation of immune response-related pathways, including chemokine signaling, cell adhesion molecule (CAMs) pathways, and cytokine-cytokine receptor interaction pathways. For this subtype, immunotherapy could be considered as a potential treatment option (69).

Shi et al. identified four subtypes of pancreatic cancer: Classical-like, Basal-like, Classical-Progenitor, and Glycomet (70). The Classical-Progenitor subtype was significantly enriched for transcription factors such as *MYC*, *MYB*, and *ATOH1*, indicating specific progenitor cell characteristics. This subtype was associated with a significantly better prognosis compared to the other subtypes. The Glycomet subtype was characterized by enrichment of pathways related to glucose metabolism.

In 2023, Zheng et al. identified two subtypes, S1 and S2, based on N6-methyladenosine (m6A) transcriptomic modifications (71). The S2 subtype exhibited a distinct m6A modification pattern compared to S1. Notably, genes associated with the Squamous subtype described by Bailey et al. and the Classical subtype defined by Collisson et al. were more enriched in S2 than in S1. Additionally, the median progression-free survival (PFS) and overall survival (OS) times of S2 were significantly shorter than those of S1. Moreover, S2 exhibited relatively lower levels of T-cell and B-cell markers compared to the S1 subtype.

In 2024, Kim et al. performed single-cell sequencing on 17 pancreatic cancer samples and identified five distinct functional subpopulations of pancreatic cancer cells (72). These included Ep_TRIM54, associated with the Classical subtype, and Ep_KRT6A, associated with the Basal-like subtype (or quasi-mesenchymal subtype). They also identified Ep_PIFO, a Basal-like cluster with unique ciliary features previously mentioned in earlier studies (43, 73), as well as Ep_MSMB, a cancer cell cluster highly associated with intraductal papillary mucinous neoplasm (IPMN). Additionally, a previously unreported cluster, Ep_VGLL1, was identified. This subpopulation exhibited basic characteristics of the Classical subtype, such as high expression of tight junction genes (*TJP1* and *OCLN*) and low expression of mesenchymal markers (VIM and S100A4), along with Basal-like subtype features, such as low expression of *SMAD4* and *GATA6*. Furthermore, their study revealed that Ep_VGLL1 spatially correlates with both Classical (Ep_TRIM54) and Basal-like (Ep_KRT6A) clusters. Based on these findings, targeting Ep_VGLL1 to block the transition from the Classical to the Basal-like subtype could become a promising new therapeutic strategy, offering novel insights and directions for the treatment of related diseases.

Several molecular markers have also been used to classify previously identified subtypes of pancreatic cancer, including *HMGA1/2* and *FGF19* (74, 75), *HAPLN1* (76), *SPDEF* (77), *SEMA3A* (78), *KRT17high*/*CXCL8*+ (79), *TEAD2* (80), *HOXA10* (81), *IRF1* (82), and *RBFOX2* (83), among others.

In recent years, with the help of advanced molecular biology techniques, a series of distinct subtypes associated with methylation modifications have been successfully identified (84, 85). These newly discovered subtypes exhibit distinct differences in the distribution of methylation sites, modification levels, and the regulatory patterns of gene expression, providing new insights into the complex pathogenic mechanisms of the disease. With the continuous innovation and development of DNA and RNA sequencing technologies, significant progress has been made in the transcriptomic classification of pancreatic cancer, which has driven the implementation of precision medicine for different transcriptomic subtypes. These transcriptomic subtypes show both commonalities and unique characteristics, yet a unified classification is still lacking. Future research needs to be extensive and systematic, aiming to accurately define transcriptomic subtypes, thereby providing molecular-level guidance for the precise treatment of pancreatic cancer.

### 2.4 Proteomic subtyping and its applications

**Figure 3:**
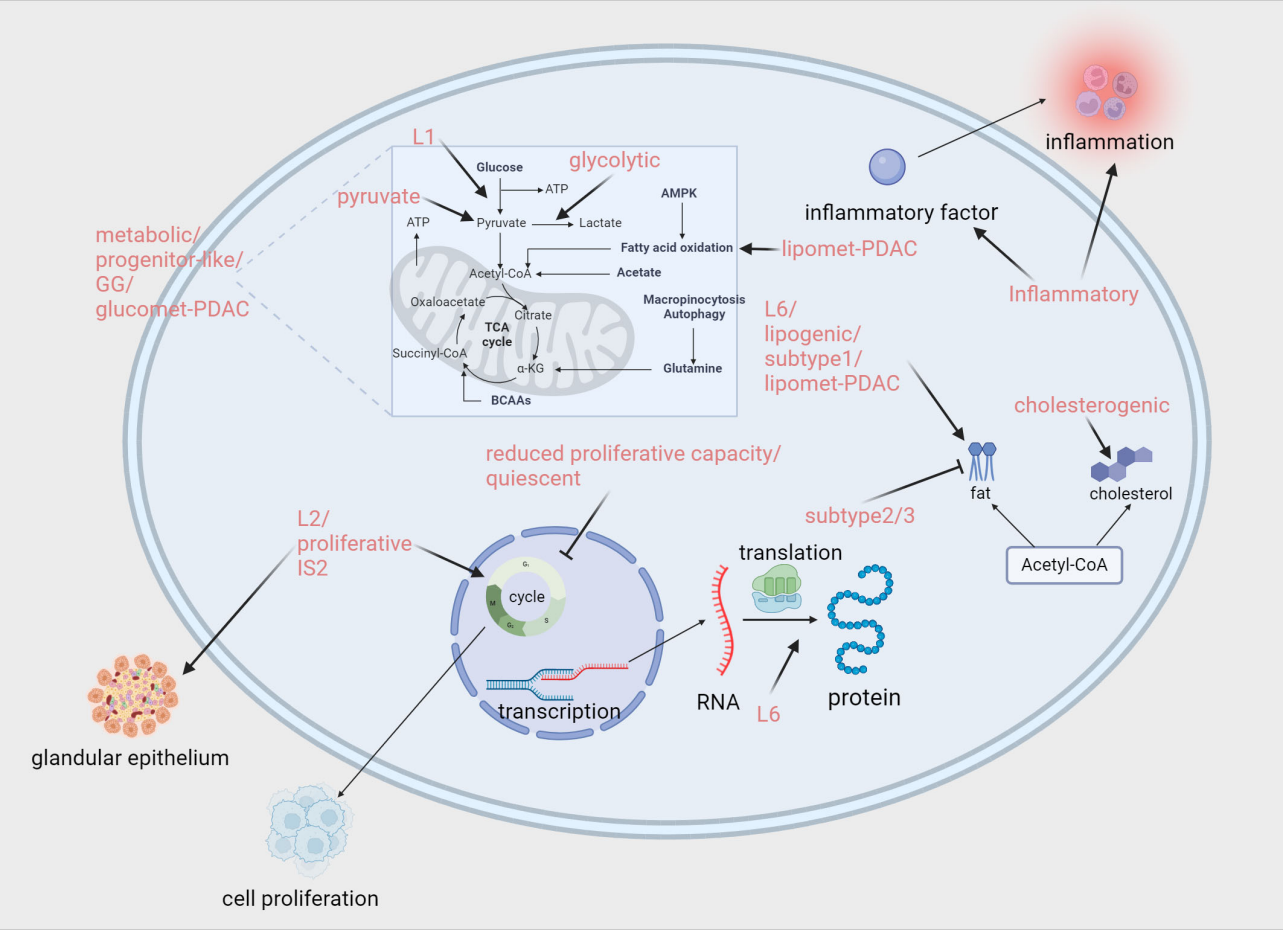
Proteomics and metabolomics-associated subtypes enriched or down-regulated pathways. Zhao (87):L1/L2/L6. Law (88): Metabolic/Progenitor-like/Proliferative/Inflammatory. Daemen (94): reduced proliferative capacity/glycolytic/lipogenic. Karasinska (96): quiescent/glycolytic/cholesterogenic. Mahajan (97): Subtype 1./Subtype 2/Subtype 3. Li (99): quiescent/pyruvate/GG. Li (101): glucomet-PDAC/lipomet-PDAC. Hyeon (92): IS2. Created in https://BioRender.com.

As we have previously discussed the genetic and transcriptomic classifications of pancreatic cancer, the next topic to address is the proteomic classification, which is downstream in the central dogma. PDAC is caused by DNA alterations, which subsequently promote tumor malignancy through RNA transcription and protein translation. Therefore, a comprehensive analysis of the functional proteomic changes in each tumor can deepen our understanding of disease progression and identify potential therapeutic targets (**Figure 3**). In recent years, advancements in various technologies have enabled the transition from identifying a limited number of proteins to conducting proteomic analyses. For PDAC, proteomic technologies have also been used to explore pathological mechanisms, diagnostic biomarkers, and therapeutic targets (86).

In 2018, Zhao et al. classified PDAC into tumor-specific subtypes (L1, L2, and L6) and stroma-specific subtypes (L3, L4, and L5) (87). L1 is enriched with carbohydrate metabolism-related gene sets and resembles the classical subtype identified by Collisson, while L6 is abundant in lipid and protein metabolism-related gene sets and aligns with Collisson’s exocrine-like subtype. These metabolism-related subtypes could potentially be targeted using metabolic drugs for therapeutic intervention. L2, characterized by epithelial and cell proliferation gene profiles associated with poor prognosis, shows similarities to Bailey’s squamous subtype. Given the high proportion of malignant epithelial cells in L2, patients with this subtype may benefit from intensified therapeutic strategies. L3 is enriched with collagen-related gene sets and is associated with poor prognosis, bearing resemblance to Bailey’s pancreatic progenitor subtype. For patients with this subtype, treatments targeting collagen may be effective. L4, which contains a variety of immune-related gene sets and is linked to relatively favorable survival outcomes, shows good responsiveness to immunotherapy. L5, enriched with neurotransmitter and insulin secretion-related gene sets and characterized by high expression of *FGFR1* pathway-associated genes, may be sensitive to neuroendocrine therapies.

In 2020, Law et al. conducted a quantitative analysis of 916 proteins from a total of 68 tissue samples to characterize four distinct PDAC liver metastasis subtypes (88): Metabolic, Progenitor-like, Proliferative, and Inflammatory. The Metabolic and Progenitor-like subtypes are characterized by an enrichment of metabolism-related proteins, including those involved in the ethanol oxidation pathway, mitochondrial fatty acid β-oxidation pathway, and retinoic acid signaling pathway. The Proliferative subtype is rich in ribonucleoproteins and Cajal body proteins, which are closely associated with translation processes, cellular proliferation, and telomere maintenance, playing a crucial role in cancer cell growth and progression. The Inflammatory subtype is enriched in proteins related to the pentose phosphate pathway, adaptive immune response, complement activation, *IL-8* production, and extracellular matrix organization. Moreover, the study revealed that the Proliferative and Inflammatory subtypes together correspond to the squamous subtype proposed by Bailey et al. In terms of chemotherapy, it is noteworthy that patients with the Metabolic and Progenitor-like subtypes demonstrated a survival advantage when treated with a combination of FOLFIRINOX and gemcitabine compared to gemcitabine alone. This finding provides valuable insights for optimizing clinical treatment strategies.

In 2021, Son et al. classified PDAC into four subtypes based on 24 protein features: stable, exocrine-like, activated, and extracellular matrix (ECM) remodeling. The stable subtype is so named because of its relatively stable disease progression and better prognosis. This subtype predominantly overlaps with the classical subtype proposed by Puleo et al., characterized by *GATA6* expression and an abundance of stromal components and pancreatic enzymes. Patients with this subtype show significantly improved survival outcomes when treated with first-line chemotherapy regimens. The exocrine-like subtype is characterized by high expression of pancreatic enzymes and is associated with the exocrine-like subtype identified by Moffitt et al. Enzyme replacement therapy may be effective for this subtype. The activated subtype is enriched in the *PI3K-AKT* and *MAPK/ERK* signaling pathways. This feature suggests that targeted therapies against receptor tyrosine kinases (RTKs) could benefit patients with this subtype. The ECM remodeling subtype is characterized by the enrichment of WNT/β-catenin and Notch signaling pathways. Targeting these two pathways may provide therapeutic benefits for patients with this subtype. Notably, the activated and ECM remodeling subtypes are highly correlated with the basal-like subtype, both of which are associated with poorer prognoses (89).

In 2022, Tong et al. conducted a comprehensive multi-omics analysis of 217 PDAC tumors and their paired non-tumor adjacent tissues, classifying PDAC into three subtypes based on proteomics: S-I, S-II, and S-III (90). The S-I subtype was associated with various metabolic processes, including the tricarboxylic acid (TCA) cycle, fatty acid metabolism, and glycolysis. The S-II subtype was closely linked to coagulation-related processes, while the S-III subtype was characterized by features such as the *ERBB2* signaling pathway and DNA damage response. Notably, these subtypes demonstrated significant overlap with those identified by Collisson et al. Specifically, the S-I subgroup overlapped with the “classical” subtype, with a concordance rate of 80.7%; the S-II subgroup overlapped with the “exocrine-like” subtype, with a concordance rate of 62.3%; and the S-III subgroup largely overlapped with the “QM-PDA” subtype, with a concordance rate of 98.1%. Furthermore, glycolysis-related proteins such as *PFKL* and *MDH2* were enriched in the S-I subtype, coagulation-related proteins such as *FGG* and *GP1BA* were enriched in the S-II subtype, and proteins like *MCM2* and *NCF1* were highly expressed in the S-III subtype. Based on these characteristics, therapeutic strategies targeting the relevant proteins and kinases within the pathways specific to each subgroup could potentially serve as viable treatment options.

In 2023, Swietlik analyzed more than 10,000 PDAC cell-derived proteins and uncovered distinct protein differences that segregate classical and mesenchymal subtypes. The classical and mesenchymal subtypes exhibited differences in secreted proteins, which were associated with immune cell recruitment and the composition of the tumor microenvironment. When interacting with macrophages, the two subtypes demonstrated distinct immunomodulatory and stromal remodeling characteristics (91).

In 2023, Hyeon performed a proteomic analysis of 171,272 peptides and 49,651 phosphopeptides derived from 196 PDAC patients from Asia, classifying PDAC into four subtypes: classical progenitor (TS1), squamous (TS2–4), immunogenic progenitor (IS1), and exocrine-like (IS2). The squamous subtype was further divided into activated stroma-enriched (TS2), invasive (TS3), and invasive-proliferative (TS4) subtypes. TS1 corresponded to the pancreatic progenitor subtype, while IS1 and IS2 corresponded to the immunogenic and exocrine-like subtypes, respectively. Tumors of the TS1 subtype were characterized by a high proportion of tumor cells, low proportions of fibroblasts and T cells, and activation of the mucin (*MUC1/4/5AC*) pathway, suggesting that targeting the mucin pathway in combination with chemotherapy could be effective. TS2–4 subtypes belong to the squamous subtype and demonstrated high proportions of tumor cells, low T cell infiltration, and enhanced activity of EMT-related pathways, such as the RhoA signaling and metalloproteinase pathways. Among these, TS4 showed the worst prognosis, potentially due to an increased proportion of polymorphonuclear myeloid-derived suppressor cells (PMN-MDSCs) that inhibit cytotoxic CD8+ T cells. These subtypes may benefit from a combination of RHOA signaling inhibitors and conventional cytotoxic chemotherapy. The squamous subtype subgroups identified through proteomics provide unique insights into therapeutic strategies for treating aggressive squamous tumors. A network model revealed increased mRNA expression of genes involved in phagocytosis, antigen presentation, and T cell receptor signaling in IS1 and IS2 subtypes. Correspondingly, proteins and phosphorylation levels within these pathways were also elevated. Additionally, IS2 exhibited increased abundance and phosphorylation levels of proteins involved in pancreatic secretion pathways, affirming its exocrine-like characteristics. Tailoring therapeutic approaches to the specific proteomic profiles of these subtypes may offer significant benefits to these patients (92).

In recent years, mass spectrometry technology has made rapid advancements, and bioinformatics engineering has seen extensive applications, providing strong impetus for the development of proteomics in the precision treatment of pancreatic ductal adenocarcinoma (PDAC). Proteomics allows for a comprehensive study of PDAC at the protein level, enabling the precise identification of protein changes closely associated with the onset and progression of the disease. This offers rich and accurate information resources for early diagnosis, the identification of therapeutic targets, and the monitoring of treatment efficacy. It is foreseeable that in future medical practice, proteomics will play an even more critical role in the precision treatment of PDAC, contributing significantly to improving patient prognosis, enhancing the effectiveness and specificity of treatment, and becoming a powerful tool in the fight against PDAC.

### 2.5 Metabolomics subtyping and applications

Metabolic reprogramming is a hallmark that regulates invasiveness and treatment response during cancer development and progression (93). In pancreatic cancer, a highly heterogeneous tumor, there are significant differences between tumor cells, which means that treatment strategies developed for a specific metabolic feature are often only effective in a subset of cancer patients. Therefore, conducting a systematic classification study of the metabolic reprogramming process in pancreatic cancer, and developing precision treatment strategies based on its unique metabolic characteristics, is of great significance and urgency (**Figure 3**). This not only helps deepen the understanding of the complex metabolic mechanisms in pancreatic cancer but also opens new avenues for achieving precision medicine in the treatment of pancreatic cancer.

In 2015, Daemen et al. conducted a quantitative analysis of 256 metabolites in 38 pancreatic cancer cell lines and identified three subtypes using non-negative matrix factorization (NMF) clustering: reduced proliferative capacity, glycolytic, and lipogenic (94). The reduced proliferative capacity subtype accounted for 34% of all lines, characterized by low levels of amino acids and carbohydrates, and a significantly longer doubling time compared to other subtypes. The glycolytic subtype exhibited elevated levels of components related to glycolysis and the serine pathway, including phosphoenolpyruvate (PEP), glyceraldehyde-3-phosphate, lactate, and serine. Additionally, this subtype demonstrated a notable feature: metabolites crucial for maintaining redox potential, such as NADH, NADP, and NADPH, were relatively low. Furthermore, genes associated with glycolysis and the pentose phosphate pathway were also expressed at higher levels in this subtype. The lipogenic subtype was enriched with various lipid metabolites, including palmitic acid, oleic acid, and palmitoleic acid, along with oxidative phosphorylation (OXPHOS) metabolites like coenzyme Q10 and coenzyme Q9. At the same time, cholesterol and lipid synthesis-related genes were upregulated in this subtype. Notably, the lipogenic subtype was associated with the epithelial (classical) subtype, while the glycolytic subtype was closely linked to the mesenchymal (QM-PDA) subtype, which aligns with the poorer prognosis associated with the glycolytic subtype. In terms of therapeutic applications, the glycolytic and lipogenic subtypes exhibited varying sensitivities to inhibitors targeting glycolysis, glutamine metabolism, lipid synthesis, and redox balance. Based on this, selecting appropriate drugs tailored to each subtype could enhance treatment efficacy for different patients, thus offering a potential strategy for personalized treatment.

In 2017, Nicolle et al. identified two metabolism-related subtypes, defined as Basal and Classical (95). Similar to the characteristics identified in previous studies, the Basal subtype exhibited stronger invasiveness and poorer prognosis, while the Classical subtype showed the opposite characteristics. Notably, this study found that the Basal subtype was associated with upregulation of genes related to the glycolytic pathway, whereas the Classical subtype exhibited a general increase in redox-related metabolites and widespread dysregulation of lipid metabolism, including a decrease in triglycerides, increased levels of fatty acids, and an increase in glycerophospholipids. Additionally, cholesterol transport proteins were significantly upregulated, and cholesterol ester levels were markedly higher, all of which indicated enhanced cholesterol uptake activity in the Classical subtype. This study suggests that targeting the metabolic characteristics of transcriptomic subtypes could be a promising and viable therapeutic approach.

In 2019, Karasinska et al. conducted a bioinformatics analysis of genomic, transcriptomic, and clinical data from a cohort of 325 PDAC cases, identifying four subtypes: Quiescent, Glycolytic, Cholesterogenic, and Mixed (96). The Quiescent subtype, as the name suggests, is characterized by low metabolic activity. Specifically, the expression of genes involved in amino acid catabolism, nucleotide metabolism, and the pentose phosphate pathway was significantly reduced, reflecting a relatively inactive metabolic state at the gene expression level. Notably, the Quiescent subtype was closely associated with the Classical subtype identified by Collisson et al. The Glycolytic subtype has distinct features, most notably a high enrichment of glycolysis-related pathways, along with amplification of the *KRAS* and *MYC* genes. Additionally, the expression levels of the mitochondrial pyruvate carriers *MPC1* and *MPC2* were significantly reduced. This subtype has been largely associated with the Basal/mesenchymal/squamous subtype in previous classifications and is clinically linked to poorer prognosis. The Cholesterogenic subtype is characterized by increased expression of MPC1 and MPC2 and enrichment of lipid metabolism-related pathways. It aligns with the pancreatic progenitor subtype and is associated with a better prognosis. The Mixed subtype combines characteristics of the aforementioned subtypes, resembling a complex “hybrid” with more diverse and complex biological features. Furthermore, an important finding of this study was that in Glycolytic PDAC cases, increasing the expression of *MPC1* and *MPC2* could potentially improve patient prognosis by promoting a transition of the tumor to a Cholesterogenic subtype.

In 2021, Mahajan studied the metabolic plasma profiles of 361 PDAC patients and identified three subtypes based on distinct lipid metabolism patterns (97). Subtype 1 exhibited elevated triglyceride levels and reduced ceramide levels. Subtype 2 showed the opposite pattern, with increased ceramide levels. Subtype 3 was characterized by significant decreases in both ceramide and triglyceride levels, along with complex fluctuations in the levels of various sphingolipid species, some of which increased and others decreased. The differences observed among these subtypes in lipid metabolism-related markers suggest that lipid metabolism plays a crucial role in the growth of pancreatic cancer. Therefore, future research may focus on investigating how lipids regulate cancer progression and exploring whether they can serve as potential targets for novel therapeutic strategies.

In 2022, Rodriguez et al. identified three distinct glycometabolic subtypes based on specific glycometabolism-related genes (98). The Fucosylated subtype was characterized by increased expression of genes involved in fucosylation (*GMDS*) and O-glycosylation (*GALNT4*). This subtype was associated with the classical/progenitor subtype identified in previous studies and correlated with better prognosis. The Basal subtype displayed elevated expression of genes encoding galectin-1 (*LGALS1*), mucin *MUC4*, and *MUC16*. It was highly correlated with mesenchymal/basal-like/squamous subtypes, which are associated with poor prognosis. The Mixed/low tumor content subtype exhibited a lower tumor cell content and was linked to the previously identified ADEX/exocrine-like subtype. The study also demonstrated that the ADEX/exocrine-like subtype frequently occurs in samples with low tumor purity.

In 2023, our team conducted proteomic and metabolomic analyses on 20 PAAD tissues and 10 normal pancreatic tissues, classifying pancreatic cancer into four TAM2-associated metabolic subtypes based on the expression profiles of pyruvate and glycolysis/gluconeogenesis (CG)-related genes: Quiescent, Pyruvate, GG (glycolysis/gluconeogenesis), and Mixed subtypes (99). The Quiescent subtype was primarily enriched in KEGG pathways related to glucose, amino acid, and lipid metabolism and characterized by serine-type endopeptidase activity, hormone secretion, zymogen activation, and immune response. The Pyruvate subtype was closely associated with the *MAPK* and *cAMP* signaling pathways and featured cation channel complexes, vesicle-mediated transport, and insulin secretion. The GG subtype showed enrichment in KEGG pathways related to glucose metabolism and was characterized by exogenous metabolic processes, detoxification, and tissue homeostasis. The Mixed subtype participated in KEGG pathways associated with immune-related biological processes and signal molecules, featuring extracellular matrix organization, antigen presentation, and serine/threonine kinase signaling pathways. Our team also investigated the efficacy of various chemotherapeutic agents across these subtypes, providing practical insights for future pancreatic cancer treatment strategies. These findings offer new directions for addressing the challenges posed by pancreatic cancer.

In 2023, a study divided 930 pancreatic cancer samples into three clusters based on the expression profiles of oxidative stress and phospholipid metabolism (OSPM)-related genes: C1 (OSPM-active), C2 (OSPM-inactive), and C3 (OSPM-normal) (100). Among these, C1 displayed the highest OSPM functional score. Importantly, the OSPM functional score was negatively correlated with tumor-infiltrating lymphocytes (TILs), T-cell co-stimulation, plasmacytoid dendritic cells, and mast cells. Moreover, C1 was characterized by the elevated expression of numerous immune checkpoint molecules, such as *HAVCR2* and *TNFSF4*. These observations suggest that C1 might be more responsive to immunotherapy due to its unique immunosuppressive microenvironment and high levels of immune checkpoint expression.

In 2023, Li et al. described the metabolomic characteristics of PDAC organoids and classified them into two distinct subtypes: glucomet-PDAC (high glucose metabolism levels) and lipomet-PDAC (high lipid metabolism levels). Glucomet-PDAC was significantly enriched in glucose metabolism, energy metabolism, and nucleotide metabolism, with pentose phosphate pathway (PPP) metabolites highly accumulated in the corresponding organoids. This subtype exhibited resistance to chemotherapy, suggesting that conventional chemotherapeutic approaches might be less effective for treating this subtype. In contrast, lipomet-PDAC was characterized by increased expression of genes associated with lipid metabolism and glycan biosynthesis. Importantly, their study identified the *GLUT1/ALDOB/G6PD* axis as a key regulator that remodels glucose metabolism in glucomet-PDAC, ultimately driving chemoresistance in this subtype. This finding offers a novel strategy to address chemoresistance in glucomet-PDAC, positioning *GLUT1* as a promising therapeutic target to overcome this challenge (101).

In recent years, significant progress has been made in the study of metabolic characteristics in pancreatic cancer, particularly in theories based on metabolomics, which have garnered widespread attention. However, due to limitations in technology and other factors, translating these research findings into effective clinical treatment strategies remains a considerable challenge. Future studies focused on metabolomics are urgently needed to foster consensus among researchers on key issues and facilitate the application of these findings in clinical practice. Only through such efforts can patients truly benefit, offering new hope in the fight against pancreatic cancer.

### 2.6 Immunomics subtyping and applications

**Figure 4:**
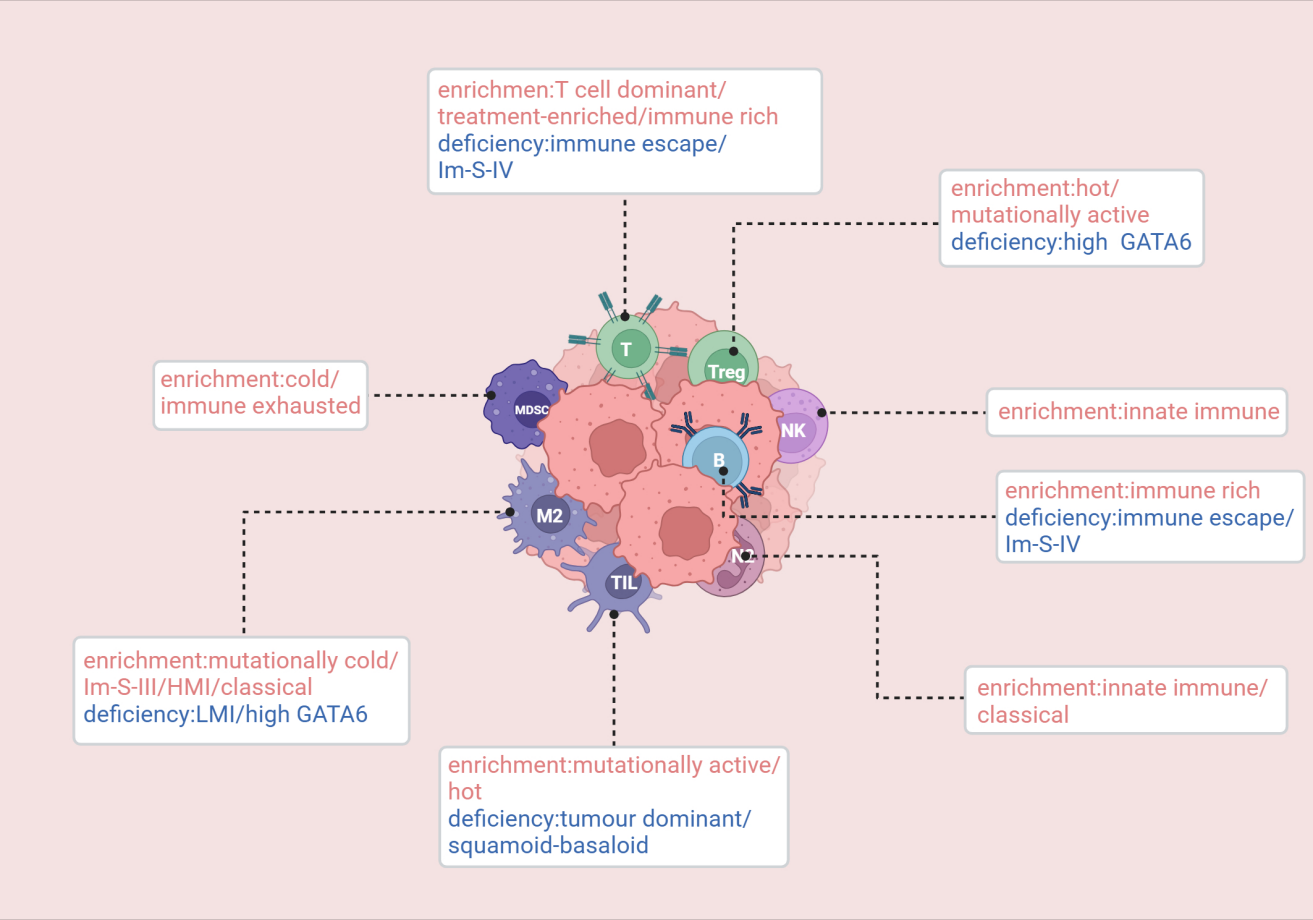
Enrichment and deficiency of immune cells in relevant immune subtypes. Knudsen (105): hot/cold/mutationally cold/mutationally active. Wartenberg (106): immune escape/immune rich/immune exhausted. de Santiago (108): innate immune/T cell dominant/tumor dominant. Hwang: treatment-enriched/squamoid-basaloid/classical. Tong (90): Im-S-IV(Metabolic-Neuron-Inflamed). Du (113): HMI/LMI. van Eijck (57): High GATA6. Created in https://BioRender.com.

In recent years, significant progress has been made in the application of immunotherapy in the field of cancer. In 2018, the Nobel Prize in Physiology or Medicine was awarded to James P. Allison and Tasuku Honjo for their discovery of immune checkpoint blockade (ICB) therapy. Although this therapy has benefited some patients with solid tumors (102), a substantial proportion of “cold” tumors show limited response to ICB therapy (103), possibly due to the diversity of immune evasion mechanisms. Currently, there are several treatment methods for pancreatic cancer, such as oncolytic viruses, modified T cells (T-cell receptor (TCR) engineering and chimeric antigen receptor (CAR) T cell therapy), CAR natural killer cell therapy, cytokine-induced killing cells, immune checkpoint inhibitors, immune modulators, cancer vaccines, and strategies targeting bone marrow cells in the contemporary context (104). However, these have shown limited effectiveness in pancreatic cancer. Many immunotherapies that are effective in other solid tumors have proven to be less effective in pancreatic cancer treatment. The current focus of immune research is to develop various immune modulation strategies to enhance T cell function, initiate or strengthen tumor-specific T cell immunity, and convert the tumor microenvironment from immune “cold” to “hot,” thereby improving the clinical treatment outlook for pancreatic cancer (103). Clearly, precision therapy is crucial at this point. Increasing evidence points to the same fact: in-depth exploration of tumor immune classification can provide valuable insights and strategies for designing more effective anti-cancer treatments, which is of great significance in improving cancer treatment outcomes and patient prognosis (**Figure 4**).

In 2017, Knudsen et al. conducted a multi-omics analysis of a cohort of 109 PDAC patients and defined four immune subtypes of PDAC: hot, cold, mutationally cold, and mutationally active (105). Both the hot and mutationally active subtypes exhibit high mutational burdens, more tumor-infiltrating lymphocytes (TILs), and peritumoral lymphocytes, along with upregulated immune checkpoints (*CTLA-4* and *PDL-1*) and regulatory T cells. However, they differ in terms of tumor-associated macrophage levels. The cold subtype is characterized by low mutational burden, low levels of immune effector and suppressive cells, mature stromal types, high stromal volume, and low numbers of neoantigens. Notably, the cold subtype is associated with increased overall survival. Given these characteristics, treatment strategies that activate the immune system, such as cancer vaccines (*MUC1*, *GVAX*) or chimeric antigen receptor T cell therapy combined with immune modulators to counteract immune suppression mechanisms, may be more suitable. The mutationally cold subtype has fewer mutations, low stromal volume, and an immature stromal type, along with high levels of *MCT4*. Its microenvironment is glycolytic and acidic, and the immune infiltration is primarily composed of macrophages.

In 2018, Wartenberg et al. identified three immune subtypes of PDAC by performing immunohistochemical staining on immune cells within the tumor microenvironment: immune escape, immune rich, and immune exhausted (106). The immune escape phenotype is characterized by low levels of T cells and B cells, with a high infiltration of *FOXP3*+ regulatory T cells (Tregs), a higher tumor budding rate, and mutations in *CDKN2A*, *SMAD4*, and *PIK3CA*. Notably, this subtype displays significantly higher levels of CA19-9, which is typically associated with poor prognosis. This subtype is strongly correlated with the previously identified squamous/mesenchymal subtype. Moreover, their study suggests that targeting the MET pathway may be effective for the immune escape subtype. The immune rich phenotype is characterized by abundant T cell and B cell infiltration, with fewer *FOXP3*+ Tregs, lower tumor budding frequency, and low mutations in *CDKN2A* and *PIK3CA*. The CA19-9 levels are the lowest among the three groups, which correlates with the best prognosis. This subtype is associated with the pancreatic progenitor subtype. The immune exhausted subtype is characterized by an immunogenic microenvironment and includes two distinct subgroups. One subgroup shows *PD-L1* expression and higher *PIK3CA* mutations, while the other is a microsatellite unstable subgroup with higher *JAK3* mutations. Interestingly, despite being classified as immune exhausted, this subtype has a relatively better overall prognosis. Furthermore, this immune exhausted subtype is highly correlated with the immunogenic subtype proposed by Bailey et al.

In 2019, Danilova et al. defined four immune subtypes of pancreatic cancer based on the expression of *CD8* and *PD-L1*: *PD-L1*+/*CD8high*, *PD-L1*+/*CD8low*, *PD-L1*-/*CD8high*, and P*D-L1*-/*CD8low* (107). *PD-L1* expression is associated with poor prognosis, while CD8+ T cell infiltration correlates with better prognosis.

In 2019, a meta-analysis based on 353 pancreatic cancer patients classified the disease into three subtypes: innate immune, T cell dominant, and tumor dominant (108). The innate immune subtype shows enrichment of natural killer (NK) cells and neutrophils, accompanied by reactive stromal proliferation. The neutrophil enrichment suggests its potential as a biomarker and clinical therapeutic target. This subtype is most strongly associated with the squamous subtype identified by Bailey et al. and is correlated with better prognosis. The T cell dominant subtype is characterized by the accumulation of many tumor-infiltrating immune subpopulations related to adaptive immunity, including activated CD8+ and CD4+ T cells as well as B cells. Moreover, genes involved in immune checkpoint inhibition, such as *CTLA4* and *BTLA*, are significantly upregulated, suggesting potential responsiveness to ICB therapy. This subtype is closely associated with previously identified “exocrine-like,” “ADEX,” and “Notch” subtypes and shows a better prognosis than the innate immune subtype. The tumor dominant subtype is characterized by a unique microenvironment with a lack of tumor-infiltrating lymphocytes, high expression of adhesion-related and epithelial genes, and high expression of *GATA6*. This subtype overlaps with the classical subtype identified by Collisson et al.

In 2020, a study integrating genomic, epigenomic, transcriptomic, and clinical data from 161 pancreatic cancer patients established four molecular subgroups (iC1/iC2/iC3/iC4) (109). The study found that the iC1 subgroup exhibited significantly higher immune scores in B cells, CD4+ T cells, neutrophils, macrophages, and dendritic cells compared to the other three subgroups. The immune characteristic scores for macrophage regulation, lymphocyte infiltration, IFN-γ response, and TGF-β response were also higher in the iC1 subgroup, suggesting its potential applicability for immune therapy.

In 2022, a clustering analysis of 176 PAAD samples from the TCGA cohort identified two CD8+ T cell-related subtypes, IC1 and IC2 (110). Among the 10 oncogenic pathways, four pathways showed significant differences between the two subtypes: cell cycle, Hippo, Nrf1, and Wnt pathways. In the IC1 subtype, the enrichment scores for these pathways were markedly higher than those in the IC2 subtype. However, the IC2 subtype displayed its own characteristics, with higher immune infiltration scores compared to IC1. Additionally, the expression levels of most immune checkpoint-related genes were significantly higher in IC2, indicating its potential suitability for immune therapy. Regarding chemotherapy, IC1 demonstrated greater sensitivity to traditional chemotherapeutic drugs.

In 2022, a study by Hwang et al. performed RNA sequencing on 43 tumor samples and successfully identified three distinct clusters: “treatment-enriched,” “squamoid-basaloid,” and “classical” (111). The “treatment-enriched” cluster was closely associated with neuroendocrine-like malignant programs, neurotrophic CAF programs, and CD8+ T cells. The “squamoid-basaloid” cluster was linked to squamous, basaloid malignant programs, and various lymphoid and myeloid cells. The “classical” cluster was associated with classical malignant programs, myofibroblast progenitor cells, adhesion CAF programs, macrophages, neutrophils, and type 2 dendritic cells.

In 2022, Tong et al. identified five tumor subgroups with distinct immune and stromal features through multi-omics analysis: Im-S-I (Stromal), Im-S-II (Monocyte-Inflamed), Im-S-III (Macrophage-Inflamed), Im-S-IV (Metabolic-Neuron-Inflamed), and Im-S-V (Metabolic-cDC-Inflamed) (90). The stromal subgroup was characterized by elevated expression of endothelial cells and stromal-associated proteins (*COL17A1*, *COL7A1*, *ITGA3*, etc.), along with upregulated *EGFR* and *ERBB2* signaling pathways. The monocyte-inflamed subgroup was characterized by high monocyte infiltration levels. The macrophage-inflamed subgroup displayed tumor-associated macrophage (TAM) infiltration, with increased expression of immune evasion markers like *HAVCR2* (*TIM-3*), and had a relatively poorer prognosis. The metabolic-neuron-inflamed subgroup was characterized by neuronal features, with upregulated neuronal receptors and channels. The metabolic-cDC-inflamed subgroup was marked by increases in cDCs, CD4+ T cells, and B cells, and pathway analysis indicated upregulation of triglyceride and lipid breakdown processes. Furthermore, antigen-presentation MHC molecules, including *HLA-E*, *HLA-DQA1*, *HLA-DQB1*, and *HLA-DRA*, were also enhanced in this subgroup. Additionally, the study explored the relationship between these subtypes and age, revealing that older patients had more immune cell infiltration than younger patients, suggesting that immune therapy may be more beneficial for older patients.

In 2022, Wang et al. used the ICGC database to classify the PDAC cohort into four subtypes: Immune-enrich-Stroma, Non-immune-Stroma, Immune-enrich-non-Stroma, and Nonimmune-non-Stroma (112). The Immune- enrich -Stroma subtype was primarily enriched in tumor immune-related molecular features. The Non- immune -Stroma subtype was characterized by features such as *PD-1* resistance, activated stroma, CAF stimulation, and normal stroma, with very low immune-related characteristics. The Immune-enrich-non-Stroma subtype was mainly enriched in tumor immune-related features, with very low expression of stromal characteristics. The Nonimmune-non-Stroma subtype exhibited few immune and stromal features.

In 2023, our team performed clustering analysis on 178 samples from the TCGA database and identified two subtypes: High TAM2 Infiltration (HMI) and Low TAM2 Infiltration (LMI) (113). The HMI cluster was characterized by genes involved in various classical tumor signaling pathways and immune processes, including *PI3K-AKT*, *NF-κB*, and *IL-17* signaling pathways, and showed a low response rate to immunotherapy, possibly related to TAM2 enrichment. However, the KEGG pathways involved in the LMI cluster were not related to tumor progression or immune response, but this subtype was sensitive to traditional chemotherapy drugs such as oxaliplatin and exhibited a better response to immunotherapy than the HMI subtype.

In 2023, Zheng et al. classified patients into two molecular subtypes of PDAC based on T cell marker genes (TMGs): Proliferative PDAC (C1) and Immune PDAC (C2) (114). The C1 group (high TMGs) was significantly enriched in cell cycle and cell proliferation-related pathways, while the C2 group (low TMGs) was enriched in immune-related pathways. The high TMGs group was significantly associated with poor overall survival (OS), suggesting that TMGs may serve as a reliable prognostic biomarker for PDAC.

In 2024, van Eijck et al. demonstrated that tumors with high *GATA6* expression exhibited reduced infiltration of immunosuppressive regulatory T cells and M2 macrophages while showing increased infiltration of immune-stimulating, antigen-presenting, and activated T cells. This study suggested that *GATA6* defines an immune-enriched phenotype, which is associated with favorable outcomes for pancreatic cancer patients undergoing preoperative treatment (57).

In 2024, George B et al. conducted a transcriptomic analysis of the tumor microenvironment (TME) in 1,657 pancreatic cancer samples from public databases and validated their findings using an independent cohort of 79 patients. Based on their analysis, the TME was classified into four subtypes: immune enriched (IE), immune enriched with fibrosis (IE/F), fibrotic (F), and immune depleted (D). The IE subtype exhibited the highest levels of anti-tumor immune components, including T cells, B cells, and natural killer cells, while also showing elevated tumor-promoting immune components, such as regulatory T cells and immune checkpoint molecules. However, the anti-tumor immune signals remained predominant. The IE/F subtype was characterized by a balanced activation of both anti-tumor and tumor-promoting immune components, with the highest activation of the WNT signaling pathway. The F subtype displayed the strongest enrichment of cancer-associated fibroblast (CAF) pathways and angiogenesis-related signals. In contrast, the D subtype exhibited the highest levels of proliferative gene signatures. Additionally, the study revealed that most lung metastases were classified as the IE subtype, whereas liver metastases were predominantly of the D subtype. The IE/F subtype showed a strong resemblance to Bailey’s ADEX subtype, while Bailey’s immunogenic subtype largely overlapped with the IE and IE/F subtypes identified in this study. The F and D TME subtypes were associated with Moffitt’s and Bailey’s classifications (basal-like and squamous subtypes, respectively) and were linked to the poorest prognosis. Furthermore, differences in surface biomarkers were observed among the TME subtypes, providing potential therapeutic implications for PDAC patients. Specifically, immune-regulatory surface biomarkers were most highly expressed in the IE and IE/F subtypes, stromal proteins were predominantly expressed in the F subtype, and signaling molecules associated with tumor invasion and survival were highly expressed in the D subtype (115).

In these studies, each subtype exhibits distinct immune characteristics, including but not limited to tumor mutational burden (TMB), *PD-1/PD-L1* levels, mismatch repair (MMR), immune checkpoint inhibitors, stromal components, and TGF-β responses. For tumor subtypes with high TMB, vaccine-based therapies may be a more suitable option, as a higher TMB indicates the presence of more genetic mutations within tumor cells, providing additional targets for vaccine-induced immune responses. In cases where a tumor subtype exhibits a higher expression of immune checkpoints, this subtype may be more sensitive to immune checkpoint blockade (ICB) therapy. Immunotherapy may be effective for subtypes with elevated expression of immune cell death regulators (5). With the growing research on immune-related mechanisms, immune subtype-based therapies are expected to bring new hope to pancreatic cancer patients in the future.

## 3 Discussion

In pancreatic cancer research, molecular subtyping plays a central role in advancing precision medicine. However, current efforts to classify pancreatic cancer face significant challenges alongside emerging opportunities. Subtypes are primarily defined based on multiple dimensions, including gene mutations, genomics, transcriptomics, proteomics, metabolomics, and immunomics. Despite these classifications, no unified consensus has been established in the medical field, and progress in translating these classifications into clinical practice remains slow, significantly hindering the development of precision medicine for pancreatic cancer.

From the perspective of multi-omics clinical trials, genomics-related trials such as NCT02869802, NCT05380414, and NCT04484636 provide crucial avenues for exploring the molecular mechanisms of pancreatic cancer. An in-depth analysis of multi-omics data from pancreatic cancer patients in the TCGA database—including gene expression profiles, methylation microarray data, and histone modification data—has led to the identification of multiple epigenetically dysregulated lncRNAs (epi - lncRNAs). These epi - lncRNAs exhibit significant genomic differences from non - epi - lncRNAs, such as increased length, a higher number of transcripts, and more exons. Further screening identified five pancreatic cancer-specific epi-lncRNA genes (AL161431.1, LINC00663, LINC00941, SNHG10, and TM4SF1-AS1), which were used to construct a prognostic model. This model demonstrated strong prognostic predictive performance across different datasets, highlighting the critical role of genomics in pancreatic cancer subtyping research (116). Additionally, immunotherapy-related trials such as NCT01072981 and NCT06370754 have injected new momentum into research on immune-related pancreatic cancer subtypes.

The integration of multi-omics approaches offers promising prospects for pancreatic cancer subtyping. However, its clinical application presents both advantages and challenges. One of its key benefits is the ability to provide a comprehensive understanding of the molecular characteristics of pancreatic cancer, enabling more precise subtyping. By integrating genomics, transcriptomics, proteomics, metabolomics, and immunomics, researchers can conduct a holistic analysis of the complex biological processes underlying pancreatic cancer. For example, the combined use of the UK Biobank and multi-omics analyses has yielded significant findings. In one study (117), researchers integrated multi-omics data from biobanks such as the UK Biobank, incorporating 4,611 genome-wide association studies (GWAS) and meta-analyses. By applying Mendelian randomization and colocalization analyses, they identified numerous disease-associated genetic loci, providing valuable insights for pancreatic cancer gene-disease association studies. Another multi-omics study (118) utilized UK Biobank data to train genetic scores for predicting multi-omics traits, conducting a phenome-wide association study that uncovered strong associations between multiple diseases and multi-omics characteristics. These findings contribute to a more comprehensive foundation for pancreatic cancer subtyping, facilitating the development of personalized treatment strategies and improving therapeutic outcomes.

Nevertheless, several challenges hinder the clinical application of multi-omics approaches. First, the acquisition and analysis of multi-omics data are costly. Genomic testing requires advanced sequencing platforms and substantial reagent investments, while proteomic and metabolomic analyses demand specialized equipment and intricate experimental procedures, limiting their widespread clinical adoption, particularly in resource-limited settings. Second, the interpretation of multi-omics data is highly complex. The vast amount of multi-dimensional data necessitates sophisticated analytical methods to extract clinically relevant insights. However, the intricate interconnections between different omics layers remain challenging to decipher, and the lack of standardized analytical approaches may lead to misinterpretation. Lastly, the standardization of multi-omics testing techniques remains insufficient. Variations in laboratory methodologies, workflows, and quality control standards contribute to inconsistencies in results, undermining the accuracy and reliability of multi-omics applications in clinical practice.

A detailed investigation has revealed that the difficulties associated with multi-omics-based subtyping are influenced by multiple complex factors. One of the most significant factors is sample purity. Pancreatic tumor samples often consist of a mixture of different cell types, and when purity is low, interference from non-tumor cells may obscure the molecular characteristics of tumor cells, leading to misclassification and a reduction in subtyping accuracy. Another important factor is the variability in analytical methodologies. Differences in experimental techniques and analytical algorithms across laboratories may lead to inconsistencies in the classification of the same tumor sample.

In addition, differences in sample composition may also interfere with subtyping. Cellular heterogeneity exists across different regions of a tumor, and variations in molecular characteristics between regions may introduce bias in classification if not adequately accounted for. Furthermore, tumor samples obtained through surgical procedures are often divided into separate sections for different types of analysis. This fragmented approach means that subtyping is frequently conducted on only part of a tumor rather than the entire tumor, which may lead to discrepancies in classification results. For example, transcriptomic analysis of one tumor section may identify a particular subtype, while proteomic analysis of another section may suggest a different classification. These findings highlight the necessity of conducting comprehensive and systematic analyses of whole tumor samples. Since tumors are complex mixtures of multiple cellular components, accurate classification requires considering them as integrated biological systems rather than isolated parts. Moreover, increasing sample size is essential to minimize the impact of individual heterogeneity. Conducting large-scale analyses across a substantial number of samples is crucial for accurately characterizing the molecular subtypes and biological patterns of pancreatic cancer.

Currently, combination therapy centered around chemotherapy remains the primary treatment for patients with advanced pancreatic cancer. As a result, many existing classification systems have been designed to predict chemotherapy response, providing a basis for clinical decision-making. By analyzing tumor gene expression profiles and proteomic features, these models can help predict a patient’s sensitivity to different chemotherapy agents, thereby guiding the selection of the most appropriate treatment regimen.

At the same time, targeted therapy and immunotherapy have emerged as key areas of research. Targeted therapies selectively inhibit specific molecular targets unique to tumor cells, while immunotherapies activate the patient’s immune system to combat cancer. These approaches are expected to expand treatment options and improve outcomes for pancreatic cancer patients. However, in clinical practice, tumor subtypes may change over the course of disease progression, which presents a significant challenge for precision medicine. A treatment strategy based on a patient’s initial tumor subtype may lose effectiveness if the tumor undergoes subtype transformation. For instance, a tumor that initially responds well to a targeted therapy may develop resistance due to changes in its molecular subtype, ultimately reducing treatment efficacy.

Given these challenges, the development of low-cost, minimally invasive subtyping techniques is essential. Liquid biopsy (119) and circulating DNA (120) analysis have shown great potential. Liquid biopsy enables real-time, dynamic monitoring of tumor molecular characteristics by detecting tumor cells, tumor-derived DNA, RNA, and proteins in a patient’s blood, providing a convenient and efficient approach for pancreatic cancer subtyping. Circulating DNA, which consists of DNA fragments released by tumor cells into the bloodstream, can be analyzed to identify tumor-specific mutations and methylation patterns, facilitating accurate subtype classification. For example, the detection of specific gene mutations in circulating DNA may enable rapid determination of a patient’s tumor subtype, thereby offering timely guidance for precision treatment.

In the future, we hope to achieve a broad consensus on subtype-based treatments for pancreatic cancer, establish unified classification standards through multi-omics integration, and provide robust guidance for clinical management. Such advancements would bring renewed hope to pancreatic cancer patients and contribute to improving their quality of life.
